# The Intersection of Radiology With Blockchain and Smart Contracts: A Perspective

**DOI:** 10.7759/cureus.46941

**Published:** 2023-10-13

**Authors:** Nima S Ghorashi, Murwarit Rahimi, Reza Sirous, Ramin Javan

**Affiliations:** 1 Department of Radiology, The George Washington University School of Medicine and Health Sciences, Washington, D.C., USA; 2 Department of Radiology and Biomedical Imaging, University of California San Francisco, San Francisco, USA

**Keywords:** smart contracts, radiology, blockchain, informatics, decentralization, non-fungible tokens, peer-to-peer communication, p2p

## Abstract

Introduction: Although blockchain technology and smart contracts are garnering attention in various sectors, their applications and familiarity within the realm of radiology remain largely unexplored. Blockchain, a decentralized digital ledger technology, offers secure, transparent, and resilient data management by distributing the verification process across a network of independent entities. This decentralized technology presents a possible solution for a range of healthcare challenges, from secure data transfer to automated verification processes. To address such challenges in the context of medical imaging, blockchain could provide different approaches, including smart contracts, machine learning algorithms, and the secure dissemination of large files among key stakeholders such as patients, healthcare providers, and institutions. This manuscript aims to explore the current attitudes and perspectives of trainees and radiologists to the utilization of blockchain technology and smart contracts in clinical radiology. Additionally, the study provides an in-depth analysis of the potential applications for incorporating blockchain into radiology.

Methods: After obtaining The George Washington University Committee on Human Research Institutional Review Board (IRB) approval, we conducted a 10-question survey among radiologists and trainees at several institutions and private practices. Surveys were created via the Google Forms application and were emailed to potential participants. Participants were asked about their current academic level (medical student, resident/fellow, academic radiologist, private practice radiologist, others), their knowledge level about the field of imaging informatics and blockchain and smart contract technologies, their level of interest in learning more about blockchain and smart contracts, and their opinion about possible applications of blockchain and smart contract in the future of medical imaging.

Results: A total of 118 survey requests were distributed; 83 were returned, reflecting a 70.3% overall response rate. Of these, 19 were sent to private practices with a 15.8% response rate (3/19), and 99 to academic centers, yielding an 80.8% response rate (80/99). The survey respondents demonstrated a strong interest and need to further understand these technologies among radiologists and trainees. This study focuses on key components of this technology as it relates to healthcare and the practice of radiology, including data storage, patient care, secure communication, and automation, as well as strengths, weaknesses, opportunities, and threats (SWOT) analysis.

Discussion: To our knowledge, this is the first study to investigate and establish a baseline for the current perspectives on the application of blockchain technology and smart contracts in clinical radiology amongst trainees and radiologists across academic and private settings. Incorporating blockchain and smart contracts technologies into the field of radiology has the potential to achieve greater efficiency, security, and patient empowerment. However, the adoption of this technology comes with challenges, such as infrastructure, interoperability, scalability, and regulatory compliance. Collaboration between radiologists, hospital administration, policymakers, technology developers, and patient advocacy organizations will help guide and advance our understanding of the potential applications of blockchain and smart contracts in radiology and healthcare.

## Introduction

Advancements in blockchain technology have led to the development of innovative use-case applications in various sectors, given its use of cryptography for the security, authenticity, transparency, and verifiability of data compared to traditional databases; however, the use of blockchain technology in healthcare and radiology in general, although quickly emerging, is at its very early inception and remains less explored and discussed [1]. These technologies may be able to help the radiology industry tackle a number of challenges, including decentralized image sharing, data privacy, direct patient-radiologist communication, and effective patient care [2].

Blockchain offers unique features to create, share, and protect massive datasets that are particularly suitable for the large DICOM files used in medical imaging [2]. More specifically, blockchain can have many clinical and research implications in medical imaging, such as the use of smart contracts, research and machine learning algorithms, autonomous authentication and verification, and cross-domain medical information and image sharing between the patient, physician, and institution [2,3]. Because of this rapidly advancing technology and its potentiality, it is important for current and future radiologists to have a general awareness of blockchain, especially with the rise of artificial intelligence in radiology, as these two technologies can complement one another in the near future and impact clinical practice.

To our knowledge, this is the first survey-based study to investigate and establish a baseline for the current perspectives on the application of blockchain technology and smart contracts in medical imaging among resident physicians, fellows, medical students, and attending physicians in the field of radiology. This paper also explores centralized vs. decentralized exchanges, privacy and encryption, oracles, non-fungible tokens (NFTs), peer-to-peer (P2P) communication, patient-physician direct communication, and details potential applications of blockchain and smart contracts in radiology. Further, it highlights the benefits and barriers to constructing a decentralized medical imaging ecosystem.

## Materials and methods

Study design: This study was designed as a multi-center, cross-sectional survey to assess the familiarity of and current perspectives on the applications of blockchain technology and smart contracts in medical imaging. Ethical approval for the study was obtained from the Institutional Review Board (IRB) of The George Washington University Committee on Human Research, and all participants were informed that their participation was voluntary and anonymous. 

Study population: The study population was recruited from the radiology departments of two academic institutions, private practice radiologists, and members of the Radiological Society of North America (RSNA) and the Society for Imaging Informatics in Medicine (SIIM). In coordination with each institution's IRB, the research team sent emails to potential participants via listserv (L-Soft, Bethesda, MD). These emails contained information about the study and an option for the recipients to consent and complete the survey. Two reminder emails were sent at two-week intervals over four weeks following the initial invitation to maximize participation.

Survey development: The survey was created by utilizing the Google Forms app. The survey consisted of three sections (Table 1). The first section had one question about the current position of the participants (medical student, resident/fellow, academic radiologist, private practice radiologist, or others). The second section contained three questions regarding the responder's knowledge level about the field of imaging informatics, blockchain and smart contract technology, and their level of interest in learning more about the role of blockchain and smart contracts in the future of medical imaging. The third section consisted of six questions regarding the participants' opinions about possible future applications of blockchain and smart contracts in patients' ownership of their images, data privacy, and security, image sharing between medical centers, assisting radiologists, assisting radiology enterprises, and assisting coding/billing. The survey employed a 5-point scale for responses, ranging from 0 (least familiar/least interested/minimal role) to 5 (most familiar/very interested/strong role). No identifying information was collected during the survey process.

**Table 1:** Survey questionnaire for study participants.

Section	Question	Response Options
Personal Information	What is your current position?	Medical student, Resident/Fellow, Academic radiologist, Private practice radiologist, Other: _______
Knowledge and Involvement	What's your level of knowledge & involvement in the field of informatics?	1 (Minimal), 2, 3 (Medium), 4, 5 (Advanced)
	What's your level of familiarity with blockchain technology and smart contracts?	1 (Minimal), 2, 3 (Medium), 4, 5 (Advanced)
	Please rate your level of interest in learning more about the future role of blockchain and smart contracts in medical imaging.	1 (Minimal), 2, 3 (Medium), 4, 5 (Strong)
Opinion on Potential Applications	In your opinion, how strongly will blockchain technology and smart contracts play a role in patients' ownership of their images?	1 (Minimal), 2, 3 (Medium), 4, 5 (Strong)
	In your opinion, how strongly will blockchain technology and smart contracts play a role in the privacy & security of data?	1 (Minimal), 2, 3 (Medium), 4, 5 (Strong)
	In your opinion, how strongly will blockchain technology and smart contracts play a role in image sharing among different centers?	1 (Minimal), 2, 3 (Medium), 4, 5 (Strong)
	In your opinion, how strongly will blockchain technology and smart contracts play a role in radiologists?	1 (Minimal), 2, 3 (Medium), 4, 5 (Strong)
	In your opinion, how strongly will blockchain technology and smart contracts play a role in radiology enterprise?	1 (Minimal), 2, 3 (Medium), 4, 5 (Strong)
	In your opinion, how strongly will blockchain technology and smart contracts play a role in coding/billing?	1 (Minimal), 2, 3 (Medium), 4, 5 (Strong)

Statistical analysis: For data analysis, Microsoft Excel 2018 was utilized. The average score for each response in sections two and three was calculated (sum of scores for each question/number of participants). Moreover, the average score for each question in sections two and three was calculated separately for each position (medical student, resident/fellow, academic radiologist, private practice radiologist).

## Results

**Figure 1:**
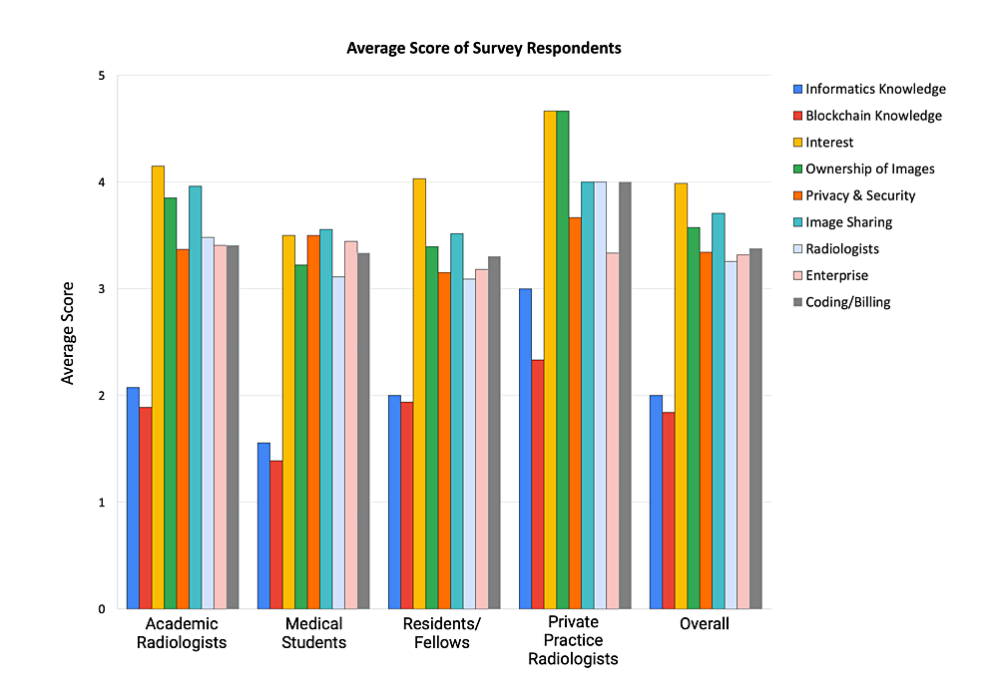
Graphical representation of survey results.

**Figure 2:**
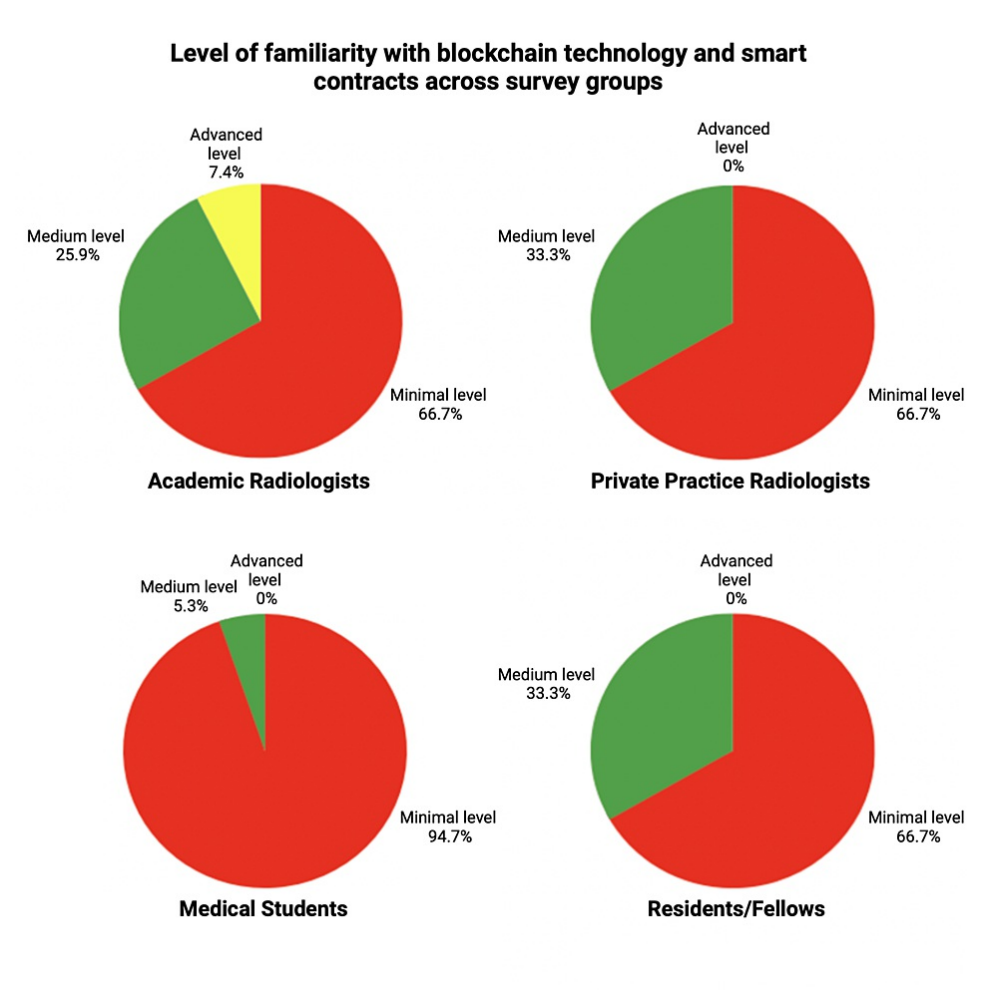
Comparison of the level of familiarity with blockchain technology and smart contracts across survey groups. Minimal level indicating a survey response of 1 or 2, Medium level indicating a survey response of 3, and Advanced level indicating a survey response of 4 or 5.

Survey responses were recorded on a scale of 1-5, with 1-2 being minimal, 3 being medium, and 4-5 being advanced and/or strong (Figure 1). A total of 118 survey requests were distributed; 83 were returned, reflecting a 70.3% overall response rate. Of these, 19 were sent to private practices with a 15.8% response rate (3/19), and 99 to academic centers, yielding an 80.8% response rate (80/99). The data demonstrate a relative lack of awareness yet a high degree of interest, particularly among the more novice trainees (i.e., medical students). Specifically, medical students’ knowledge of image sharing and privacy and security, as well as their interest in learning more about the future role of blockchain and smart contracts in medical imaging, was an average of 3.5 out of 5. This was considerably less of an interest in comparison to private practice and academic radiologists. All survey response groups showed a low average recording of 1.56 out of 5 (medical students) to 3 out of 5 (private practice radiologists) when asked about their knowledge and involvement with informatics. Only academic radiologists reported a representation (7.4%) of advanced familiarity with blockchain and smart contracts, whereas other survey groups had none (Figure 2). Notably, there is a lack of overall knowledge in informatics and blockchain across all survey groups. Despite this, survey groups overwhelmingly believed that blockchain technology and smart contracts have the potential to play a role in patient ownership of images, privacy, and security.

## Discussion

Our survey data, which revealed a high level of interest among medical students, suggests an interest in learning more about emerging technologies and practices related to smart contracts and blockchain technology as it relates to the field of radiology. However, their relative lack of awareness indicates a gap in the current educational curriculum for radiology trainees, given the rapid advancement and novelty of these technologies. Private practice and academic radiologists showing higher interest could be due to their exposure to real-world applications and challenges that can potentially be circumvented by the future implementation of these technologies. Interestingly, despite the general lack of knowledge in informatics and blockchain across all survey groups, there was a collective belief in the potential for blockchain technology and smart contracts to significantly impact patient ownership of images, privacy, and security.

Several limitations should be considered when interpreting these findings. First, the study is subject to selection bias, as participants were limited to specific institutions and attendees at a national conference meeting. Second, the use of a 5-point scale limits the granularity of the results, and the cross-sectional nature of the study limits the assessment of longitudinal changes in attitudes or knowledge about this topic. Lastly, the relatively small sample size and private practice response rate, although significantly higher response rate for academic centers, could impact the reliability and generalizability of the results.

We provide a strengths, weaknesses, opportunities, and threats (SWOT) analysis discussing the internal strengths and weaknesses as well as the external opportunities and threats in relation to blockchain technologies (Table 2). Further, there are many categories and ecosystems to consider within blockchain technology as they may pertain to the field of radiology. Essentially, a blockchain ecosystem is a network of participants in a blockchain network with similar objectives, relationships, and processes. The network is capable of developing and transferring values effectively and various ecosystems can interplay with one another to ensure the proper functioning of blockchain transactions. Here, we discuss some of these categories and delve into some detail regarding their respective pros and cons (Table 3).

**Table 2:** SWOT analysis of blockchain and smart contract in radiology.

Strengths (S)	Weaknesses (W)
Secure and tamper-proof data storage	Resource-intensive infrastructure
Patient empowerment and control over medical data	Potential delays in updating/accessing records
Automation of administrative tasks through smart contracts	Vulnerabilities to cyberattacks and human errors
Enhanced security of digital assets via NFTs	Complexities in data management and sharing
Decentralized data sharing	Governance and accountability challenges
Improved data privacy	Scalability concerns
Opportunities (O)	Threats (T)
Collaboration of radiologists, policymakers & tech	Regulatory compliance challenges
Development of best practices and governance models	Ethical and legal concerns surrounding NFTs
Integration with existing healthcare systems	Resistance to change in traditional workflows
Patient privacy advocacy	Infrastructure-related concerns
Educational initiatives	Information overload
Consortium blockchains	Fragmented communication channels

**Table 3:** Pros and cons of blockchain technology across discussion categories.

Category	Pros	Cons
Data Storage & Management	Secure, tamper-proof ledger for storing patient data	Resource-intensive, potential delays in updating/accessing records
Centralization vs Decentralization	Seamless data sharing, no single point of failure	Challenges in governance, accountability, and regulatory compliance
Patient Empowerment & Personalized Care	Patient control over medical data, P2P communication, direct patient-physician communication	Information overload, fragmented communication channels, coordination issues
Security, Privacy & Collaboration	Improved data privacy, encryption, and secure communication	Vulnerable to cyberattacks, human errors, complexities in data management and sharing
Automation & Efficiency through Smart Contracts	Automation of administrative tasks, efficient billing and insurance processing	Reliance on oracles, potential for erroneous or manipulated data, new points of vulnerability
NFTs	Enhanced security and trustworthiness of digital assets, proof of ownership	Ethical and legal concerns, potential commodification and exploitation of patient data for financial gain

We will now explore data storage, centralized vs. decentralized databases, privacy and encryption, smart contracts and oracles, NFTs, P2P communication, and patient-physician direct communication. Further, we will highlight the benefits and barriers to constructing a decentralized medical imaging ecosystem.

Data Storage and Management

Secure data management and storage are among the more discussed applications of blockchain technology in radiology [3]. Electronic medical records (EMRs), a common form of traditional data storage, are prone to security flaws and data manipulation [4]. A more secure option is provided by blockchain, which offers an immutable, tamper-proof ledger for storing patient data, including medical records and images [5]. The implementation of blockchain-based data storage, however, can be resource-intensive, increasing energy consumption and potentially slowing down the speed at which transactions are processed as the network expands. Delays in updating and accessing medical records could be harmful in time-sensitive clinical situations [6] due to the requirement for consensus among network users.

Implementing blockchain-based data storage can increase data integrity, audibility, and privacy [3]. Furthermore, radiologists, patients, and referring doctors can utilize blockchain technology's decentralized nature to more conveniently share data seamlessly across facilities [2]. Additionally, blockchain technology circumvents some of the limits associated with traditional data storage and transmission methods by enabling the safe sharing of large medical image files [7].

This technology can also allow for archiving patient information. It can streamline informed consent via cryptographic validation. For example, Benchoufi et al. designed a proof-of-concept protocol consisting of time-stamping each step of the patient’s informed consent form collection in clinical trials using blockchain [8]. Aside from clinical trials, this concept can be redirected to radiologic procedures. This may enhance individualized data sharing across different networks due to the enhanced ability to store large amounts of data without piracy concerns.

Centralized vs Decentralized Database Technology

Unlike conventional centralized database technology, blockchain technology offers a decentralized method for how medical data is stored, received, and exchanged. Decentralized systems share the management and control of data across several nodes or entities, as opposed to centralized systems, where a single authority or institution does so. One main disadvantage of centralization is the risk of a single node of a server becoming non-functional, leading to the inoperability of data across that network. Centralization does not require a large amount of investment in data centers, allowing it to be a less expensive alternative. For instance, blockchain technology can significantly benefit the field of radiology by allowing imaging centers to store and exchange data without the interruption of larger-level organizations or intermediaries. Further, this can provide this foundation for decentralized exchange of data in radiology, giving patients, radiologists, and other stakeholders the ability to take part in decision-making and to safely share their medical information [2].

Although the decentralized nature of blockchain technology might enable smooth data exchange among various parties, it may also bring up difficulties with accountability and governance [9]. A decentralized system may result in conflicts and inconsistent data handling procedures since no one authority oversees monitoring data management and security [6]. Additionally, the absence of a central authority may make it more difficult to police data privacy and regulatory compliance norms, increasing the danger to patient security and privacy [10].

Patient Empowerment and Personalized Care

By enabling patients to take control of their medical records and securely sharing them with the healthcare providers of their choice, blockchain technology can play a critical role in patient empowerment [11]. Blockchain can promote confidence and openness in the healthcare sector by allowing individuals to own their data [7]. Radiologists may access a patient's whole medical history, including radiological images and reports from numerous sources in real time due to the use of blockchain technology [12]. This may result in more timely, subspecialized, and accurate diagnoses and customized treatment regimens, which will eventually improve patient outcomes [7].

Security, Privacy, and Collaboration

Although blockchain was initially designed to store minimal personal data as an anonymous way of authenticating transactions without disclosing a party’s identity or using a trusted intermediary, newer networks are utilizing blockchain technology to maintain a level of transparency, which can raise concern for health data protection. Blockchain-based EMRs or picture archiving and communication systems (PACS) imaging databases can be customized to adjust for various levels of privacy. Patients have pre-established rights afforded to them across medical systems, and these rights should continue to be supported in the advent of this new technology.

Blockchain technology can allow healthcare professionals and patients to communicate more securely with additional functionality integrated into the digital health platform [2]. Blockchain networks offer P2P technology that enables direct sharing of sensitive medical data without the use of an intermediary entity, potentially mitigating data breaches [11]. This technology incorporates advanced cryptographic methods to protect patient data and enable improved data transfer between authorized users [13]. By improving confidence between patients and healthcare professionals, this added layer of security may promote a wider use of digital health platforms [14]. Further, blockchain can promote a more integrated healthcare system by offering a secure platform for sharing large medical imaging and data files, facilitating cross-institutional collaboration [1].

While blockchain technology can enhance privacy and encryption in the field of radiology, it is not impervious to security flaws. While cryptographic methods can be employed to protect patient data, this technology may not be sufficient to thwart human mistakes or sophisticated cyberattacks [15]. Encryption improves data security, but it can also complicate data administration and sharing, which can hinder the easy exchange of information between healthcare providers [15].

Automation and Efficiency Through Smart Contracts and Oracles

Smart contracts are programmed, self-executing agreements that take effect when certain criteria are satisfied [1]. Smart contracts can be used in radiology to automate several administrative tasks, including billing, filing insurance claims, and scheduling appointments [5]. Smart contracts can assist in reducing administrative overhead, limit human error, and increase efficiency by automating these processes [3]. Additionally, the openness and imperviousness of smart contracts might increase trust and lessen conflicts between different players in the radiological ecosystem [4].

Oracles are external data sources that can provide smart contracts access to information from the actual world. Oracles may be utilized in radiology to provide pertinent data to smart contracts for process automation, such as diagnostic imaging findings or patient medical history [5]. Radiology clinics may expedite a variety of administrative activities, increase productivity, and lessen the chance of human mistakes by combining oracles with smart contracts [3]. A smart contract's outcome might be defective if an oracle gives it the wrong data, which could result in erroneous billing or the rejection of insurance claims [14].

Radiologists may be able to transmit real-time data consisting of patients’ images, for example, and transfer these across networks that are within the same chain. Similar healthcare networks would be allowed to transmit and relay data through a smart contract. Due to the security model of blockchain networks, smart contracts are isolated from the external world and cannot natively connect to off-chain systems. Another facet of smart contracts is their use for credentialing, licensure, and facilitating contracts and reimbursements. This information can be easily stored and quickly accessed. Smart contracts can also provide rapid and secure reporting and billing. Pertinent data from the medical record can be pulled that meets the predetermined criteria, allowing authorization to rapidly occur.

NFTs in Radiology

NFTs are unique digital assets that can be used to represent ownership or provenance of various items, including digital art and collectibles. They all have unique identification codes that differentiate them with their unique value. In radiology, NFTs can be employed as a unique identifier to represent the ownership of medical imaging studies with associated reports, ensuring that they are securely stored, easily transferable, and resistant to tampering or forgery [16]. More specifically, ownership could potentially involve three parties: the patient, the radiologist, and the institution. First, the patient may own their medical images and reports as they pertain to their personal medical history and information and may have the right to control the sharing of such information. Second, radiologists may also be considered the owners of their intellectual interpretation and analysis embedded in the radiological reports and studies. While also enhancing the security and trustworthiness of digital assets, radiologists would also potentially have new revenue streams through the monetization of digital medical images [6]. Other advantages of NFTs include enhanced safety and access to stored data. The decentralized nature of this network may allow for better access to studies for research purposes and provide teaching opportunities across practices and institutions. In an EMR system, no one imaging study stored on an entire PACS network is ascribed the same accession number; thus, each study has a unique identifier. Therefore, each imaging study can be considered an NFT. NFT technology on the blockchain can enhance the sharing of studies between institutions.

While NFTs can enhance the security and trustworthiness of digital assets in radiology, their adoption may also raise ethical and legal concerns related to the ownership and commercialization of medical images and data [17]. The use of NFTs to represent ownership of medical images could potentially lead to the commoditization of patient data, resulting in privacy violations and the exploitation of sensitive health information for financial gain [18].

While smart contracts and blockchain technology have tremendous potential in radiology, there are still obstacles to their general use. These include the necessity for a strong infrastructure to support these technologies, regulatory compliance, interoperability with current systems, and scalability. It is essential for radiologists to stay up to date on the most recent technological advancements and to participate in continuing debates regarding the possible uses and restrictions of blockchain technology and smart contracts in their industry [1]. This will aid radiologists in making well-informed choices on the adoption and use of these technologies in their practice.

The radiology community should work with other participants in the healthcare ecosystem, such as policymakers, technology developers, and patient advocacy organizations to further explore the potential benefits and address key challenges associated with the adoption of blockchain and smart contract technologies [12]. Of note, this technology must continue to be improved, and best practices must be established for their use in radiology to address current weaknesses and limitations. Consortia blockchains, where a set of reputable healthcare institutions work together on data management and governance, could be one potential solution [19]. This strategy can maintain the advantages of decentralization while ensuring data security and privacy.

Looking ahead, large language models (LLM) such as ChatGPT can be instrumental in integrating blockchain and smart contract technologies in radiology [18]. These models' natural language processing abilities can be used to automate a variety of tasks, including the reading of medical reports, gathering of pertinent data, and subsequent production of structured data [20]. To share and retrieve medical records more effectively, these structured data can subsequently be safely kept on a blockchain [11]. Additionally, radiology workflows can be improved to enhance decision-making, administrative load reduction, and patient care by the integration of LLMs into smart contracts [19]. LLMs can be employed to enable direct patient-physician contact using decentralized platforms driven by blockchain technology in addition to automating tasks [21]. These can serve as middlemen, enabling an effective information exchange between patients and radiologists while protecting the confidentiality and security of sensitive patient data [8]. Users may also be rewarded, especially in terms of using their unused GPU, or graphics processing unit, power to help LLMs properly function. Furthermore, ChatGPT-like models can help with the creation and application of intelligent oracles for smart contracts, guaranteeing accurate data input and trustworthy contract execution [9]. The discipline of radiology can possibly reach new levels of efficiency, security, and patient empowerment by utilizing LLMs in conjunction with blockchain and smart contracts [20]. Recently, a new cryptocurrency named Worldcoin has been developed by Sam Altman, the CEO OpenAI, where the ultimate goal is for it to serve as the basis for universal basic income, in light of the rapid advancements in AI and the potential for significant portion of jobs being performed by AI [22]. As of July 13, 2023, two million users have provided their biometric data mainly a scan of their iris, which serves as proof for being a human rather than a bot. Lastly, considering the environmental implications, it is essential to acknowledge and address the unsustainable energy consumption associated with blockchain technology, especially for possible healthcare applications such as radiology [23]. While traditional blockchains are notorious for their high energy demands, moving forward, selecting energy-efficient models is crucial.

## Conclusions

The survey results highlight both an existing gap in knowledge of yet a strong interest in blockchain and smart contracts within the field of radiology. Addressing this educational gap could have significant implications for the adoption of new technologies that have the potential to positively impact patient care, privacy, and security. Future studies could delve deeper into the reasons behind the differences in the levels of interest and knowledge among different groups and could investigate the potential for educational interventions to improve familiarity and competency in these key areas, as these technologies are rapidly expanding.

For radiologists, having a basic understanding of blockchain and smart contracts and staying up-to-date is, therefore, beneficial. By familiarizing themselves with these technologies, radiologists can make informed decisions about how they could either be beneficial and/or harmful in various scenarios. It is important for radiologists to be aware of the applications to stay ahead of the curve. This proactive approach may identify potential pitfalls ahead of time and discover innovative use cases for blockchain and smart contracts. By tackling issues related to data storage, patient empowerment, secure communication, and process automation, blockchain technology and smart contracts have the potential to greatly influence the practice of radiology.
